# Polarization-diversity backscatter communication based on programmable information metasurface

**DOI:** 10.1016/j.isci.2025.114577

**Published:** 2025-12-30

**Authors:** Guoliang Luo, Xiangjin Ma, Jiaqi Han, Lihao Zhu, Rui Li, Haixia Liu, Long Li

**Affiliations:** 1 Key Laboratory of High-Speed Circuit Design and EMC of Ministry of Education, School of Electronic Engineering, Xidian University, Xi’an 710071, China

**Keywords:** Applied sciences, Engineering, devices

## Abstract

Backscatter communication (BC) is a low-power technology that transmits data by reflecting ambient RF signals. Conventional BC, based on antenna impedance modulation, suffers from low data rates and poor interference resistance. Programmable information metasurface (IMS) offers a breakthrough by enabling precise wave control. This article presents a PIN-diode-based IMS for polarization-diversity backscatter transmission. It performs full-space secondary modulation, converting horizontally polarized incident waves into ±45° polarized signals. Operating at 5 GHz, the system uses two polarization channels with BPSK and BASK modulation to transmit the same data, achieving 2 Mbps with improved reliability through diversity. The proposed system exhibits low power consumption, strong interference resistance, and high stability, making it suitable for IoT applications such as smart healthcare and environmental monitoring.

## Highlights

• A polarization diversity backscatter communication system based on information metasurfaces
• The system can achieve distinct information modulation schemes in two polarization directions
• The information metasurface system significantly enhances anti-interference performance

## Graphical abstract

**Figure:**
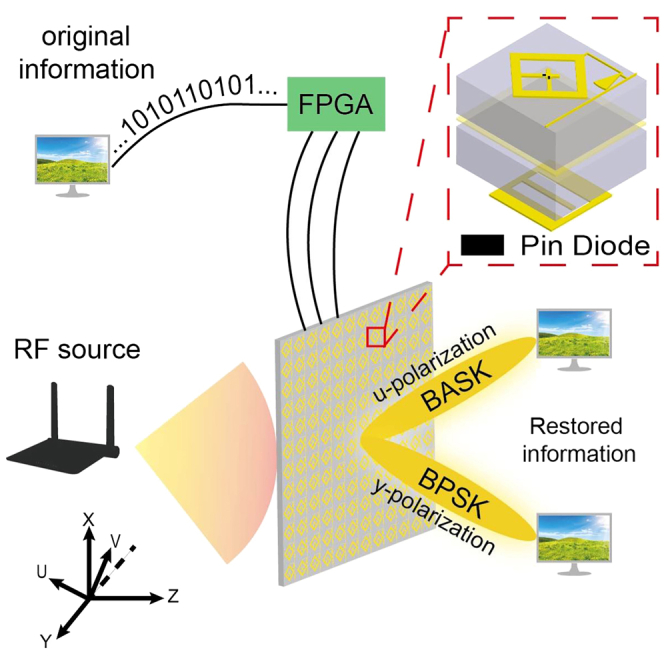

## Introduction

With the rapid development of information technology, widespread deployment of the IoT and smart devices is fundamentally transforming human lifestyles and work patterns.^1^^,^^2^^,^^3^ The IoT enables real-time data transmission and collaborative intelligence by interconnecting massive networks of intelligent devices and sensors. However, escalating device density imposes growing demands on network connectivity and communication efficiency, positioning low-power, high-efficiency, and scalable communication technologies as critical research priorities.

To address these challenges, low-power wireless communication emerges as a critical approach for IoT systems. Among various low-power techniques, backscatter communication (BC) has gained significant research traction in IoT applications due to its inherent advantages of ultra-low energy consumption, minimal cost, and robust adaptability.^4^^,^^5^^,^^6^ BC modulates and reflects ambient electromagnetic waves without requiring active transmitters, making it ideal for power-constrained scenarios such as sensor networks, smart homes, and remote monitoring. Despite its energy efficiency, conventional BC systems face practical limitations. Traditional implementations relying on simple reflectors suffer from compromised signal integrity, severe interference susceptibility, and restricted transmission range.^7^^,^^8^^,^^9^ In complex environments, multipath fading and signal attenuation further degrade performance. Consequently, enhancing communication quality, interference resilience, and operational range constitutes a current research imperative.

Recent years have witnessed extensive research attention on metasurface technology—a novel paradigm for electromagnetic wave manipulation. As artificially engineered materials with subwavelength structures, metasurfaces dynamically control wave propagation characteristics,^10^^,^^11^^,^^12^^,^^13^ including reflection, refraction, and transmission,^14^^,^^15^^,^^16^^,^^17^ through precise adjustments of unit cell geometry or electrical properties. Their ultra-thin profile, lightweight construction, power efficiency, and high performance have enabled breakthroughs in antenna design,^18^^,^^19^^,^^20^^,^^21^ beamforming,^22^^,^^23^^,^^24^^,^^25^^,^^26^^,^^27^ stealth technology,^28^^,^^29^^,^^30^^,^^31^ holographic imaging display,^32^ polarization conversion,^33^ harmonic control and generation,^34^ and wireless power transfer.^35^^,^^36^^,^^37^^,^^38^ Particularly in wireless communications, metasurfaces facilitate unprecedented flexibility in wavefront control.^39^^,^^40^^,^^41^ Integrating metasurfaces with BC systems offers transformative solutions to overcome interference, attenuation, and range limitations in conventional approaches.^42^ Metasurfaces enable precise wave manipulation, optimized signal paths, and effective mitigation of signal degradation.^43^ This synergy not only elevates communication robustness but also reduces power consumption while enhancing system scalability and adaptability, establishing metasurface-enabled backscatter communication as a frontier in wireless research. For example, ref.^44^ develops a polarization-insensitive metasurface-based BC system operating independently of polarization or incident angle; however, its reliance on conventional BASK modulation renders it vulnerable to environmental interference. Ref.^45^ proposes a Quadrature Phase Shift Keying (QPSK) scheme that requires each unit cell to be equipped with two varactor diodes, resulting in high cost and increased control complexity, and can only operate in one polarization direction. For polarization modulation (PoM), ref.^46^ presents a programmable metasurface in PoM and experimentally demonstrates a prototype of the PoM transceiver for wireless communications, but the polarization discrimination antenna has a complex design and is limited to one channel. Ref.^47^ introduces a Solar-Powered Light-Modulated Microwave Programmable Metasurface (SLMPM), which integrates a photovoltaic module to simultaneously harvest solar energy and acquire information from modulated light, enabling reliable light-to-microwave transmission, self-sustained power supply, and a hybrid communication system for real-time image transmission. However, this system has a complex structure, with a maximum communication rate of merely 234.375 kbps under QPSK modulation.

This work presents a polarization-diversity backscatter transmitter leveraging a programmable information metasurface. The metasurface converts horizontally polarized incident waves into transmitted waves with programmable +45° or −45° polarization states via PIN diode control. This ±45° polarization conversion intrinsically suppresses direct interference and mitigates multipath effects. When received under ±45° polarization, the metasurface operates as a BASK modulator; under horizontal polarization reception, it functions as a BPSK modulator. Unlike conventional single-channel transmitters, our architecture transmits identical information through two polarization channels, significantly enhancing transmission reliability and stability. Strategic placement of transmitter and receiver on opposite metasurface sides further minimizes direct coupling. The proposed programmable metasurface solution offers dynamic reconfigurability, ultralow power consumption, and seamless integrability, demonstrating strong potential for next-generation IoT applications.

## Results

**Figure 1:**
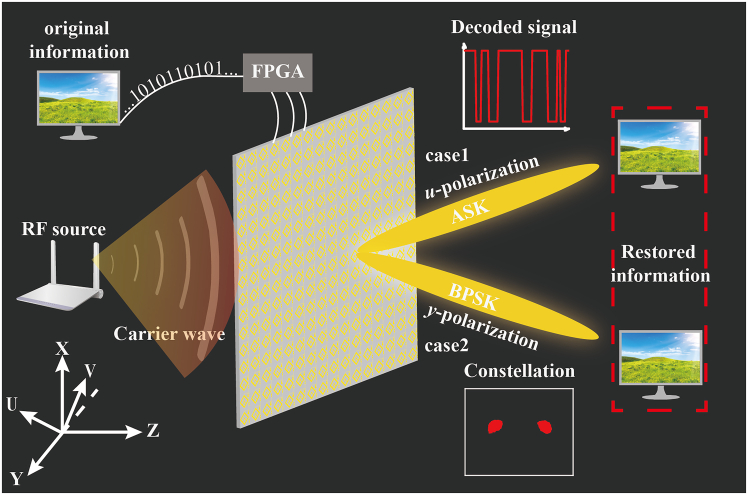
Proposed IMS-based polarization-diversity backscatter communication system

The schematic diagram of the polarization-diversity backscatter communication system utilizing IMS is illustrated in Figure 1. The BC system integrates an IMS, ambient scattered signals, and an FPGA control circuit that stores the image bitstreams. The IMS is equipped with two PIN diodes welded in reverse, which means that these two diodes cannot be turned on simultaneously. When the FPGA outputs a high level (+5V), the state of the diodes is 0/1 (0 represents off and 1 represents on), and at this time, the transmitted wave exhibits a polarization of +45° orientation. Similarly, when the FPGA outputs a low level (-5V), the transmitted wave exhibits a polarization of −45° orientation. In order for the system to transmit a picture, the picture needs to be converted into a bitstream. Based on the mapping relation between the bitstreams and control signal levels, the bitstreams are converted into the control signals to be stored in the FPGA. By periodically switching the level through the FPGA, the transmitted wave’s polarization state undergoes periodic changes. When the receiving antenna is in the +45° polarization (or −45°) state, the amplitude of the received signal will change periodically, that is, the metasurface performs BASK modulation on the incident signal. When the receiving antenna is in the horizontal polarization state (y-polarization), the amplitude of the received signal remains basically unchanged, but the phase difference between different states is 180°, that is, the metasurface performs BPSK modulation on the incident signal. The original picture information can be restored by performing the corresponding decoding operation on the received signal.

## Design principle of the programmable information metasurface

To describe the polarization conversion characteristics of the IMS more accurately, we introduce the transmission coefficient matrix *T*^*LP*^, as shown in Equation 1. For a transmissive metasurface under orthogonally linearly polarized (LP) incident waves, denoted as $E_{x}^{i}$ and $E_{y}^{i}$. The incident wave and the transmitted wave ($E_{x}^{o}$ and $E_{y}^{o}$) are related by the transformation matrix *T*^*LP*^, and the formula is as follows:

(Equation 1) $$\left(\begin{array}{l} E_{x}^{o}\\ E_{y}^{o} \end{array}\right)=T^{LP}\left(\begin{array}{l} E_{x}^{i}\\ E_{y}^{i} \end{array}\right)=\left(\begin{array}{cc} T_{xx} & T_{xy}\\ T_{yx} & T_{yy} \end{array}\right)\left(\begin{array}{l} E_{x}^{i}\\ E_{y}^{i} \end{array}\right)$$

**Figure 2:**
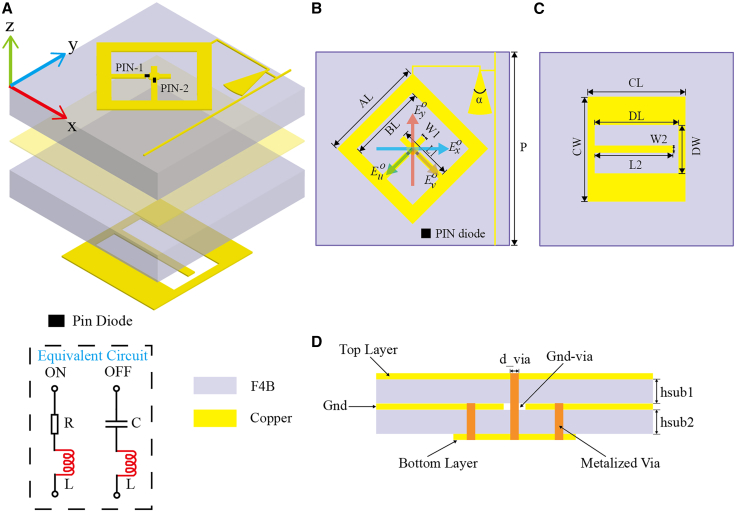
Geometric structure of the IMS (A) Exploded view. (B) Element top view. (C) Element bottom view. (D) Element side view.

Where *T*_*xx*_, *T*_*xy*_, *T*_*yx*_, and *T*_*yy*_ are the transmission coefficients (the first subscript indicates the polarization of the transmitted field, and the second subscript indicates the polarization of the incident field). For *u/v*-polarized transmitted waves, according to Figure 2B, the transmission matrix can be expressed as:

(Equation 2) $$\left(\begin{array}{l} E_{u}^{o}\\ E_{v}^{o} \end{array}\right)=\frac{\sqrt{2}}{2}\left(\begin{array}{cc} 1 & 1\\ 1 & -1 \end{array}\right)\left(\begin{array}{l} E_{x}^{o}\\ E_{y}^{o} \end{array}\right)$$

Therefore, when the incident waves are *x*-polarized and *y*-polarized, and the transmitted waves are *u*-polarized and *v*-polarized, the polarization conversion matrix is:

(Equation 3) $$\left(\begin{array}{l} E_{u}^{o}\\ E_{v}^{o} \end{array}\right)=\frac{\sqrt{2}}{2}\left(\begin{array}{cc} 1 & 1\\ 1 & -1 \end{array}\right)\left(\begin{array}{cc} T_{xx} & T_{xy}\\ T_{yx} & T_{yy} \end{array}\right)\left(\begin{array}{l} E_{x}^{i}\\ E_{y}^{i} \end{array}\right)=\left(\begin{array}{cc} T_{ux} & T_{uy}\\ T_{vx} & T_{vy} \end{array}\right)\left(\begin{array}{l} E_{x}^{i}\\ E_{y}^{i} \end{array}\right)$$

A programmable IMS composed of three metallic layers is presented in Figure 2A. The metal pattern at the bottom consists of a U-shaped slot. This slot functions to collect energy from incident waves and transfer it to the upper layer through a metal aperture. As depicted in Figure 2B, the top metal pattern is made up of a 45°-rotated square loop patch and a cross - shaped metal strip. Both metal patterns are etched onto F4B substrates (i.e., Substrate #1 and Substrate #2; εr = 3.5, tanδ = 0.001) with a substrate thickness of 3.048 mm. A 35 - μm - thick copper foil is used to separate the two substrate layers. Two PIN diodes (model: SMP1340_040lf) are inserted into the slots with opposite polarity directions. During the simulation process, the electrical parameters of the PIN diodes are carefully considered (*R*_*on*_ = 0.85 Ω, *C*_*off*_ = 0.21 pF, *L*_*on*_ = *L*_*off*_ = 0.45 nH). It is important to note that these two PIN diodes cannot operate in the same state simultaneously; one must be in the forward-bias state, while the other is in the reverse-bias state. The centers of the top and bottom patches are connected via a metallized through - hole. To facilitate the design of the bias line, two metallized blind vias are placed at the weak-field points of the bottom patch and connected to the ground, ensuring that radio frequency (RF) and direct current (DC) signals have the same ground potential. The top square loop is linked to the DC bias line via a fan-shaped branch, thus minimizing the impact of the RF signal on the DC signal. The optimized geometric parameters of the element are listed later in discussion: *p* = 27, *AL* = 14.9, *BL* = 11.1, *L1* = 7, *W1* = 0.8, *α* = 30(deg), *CL* = 13.7, *DL* = 11.7, *CW* = 14.7, *DW* = 6.8, *L2* = 10.9, and *W2* = 1 in millimeters.

**Figure 3:**
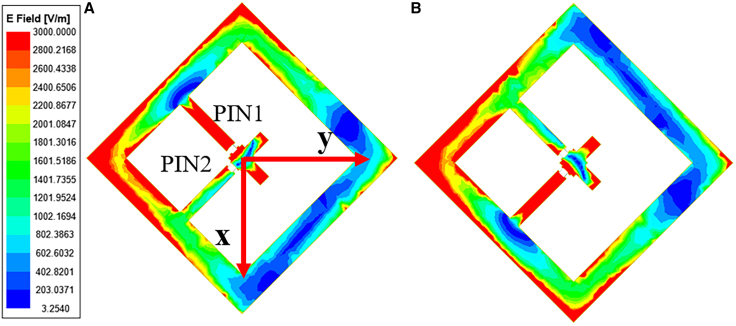
Electric field distributions in the IMS (A) PIN diode in the 0/1 state. (B) PIN diode in the 1/0 state.

Commercial software Ansys Electronics was utilized to execute full-wave simulations, integrating periodic boundary conditions and Floquet ports as excitation sources. An *x*-polarized wave propagating along the +*z* direction was used as the incident wave. Upon transmission through the metasurface, this wave is converted into ±45° polarized waves (i.e., *u*-polarized and *v*-polarized states). The two operational states of the metasurface unit are designated as 0/1 and 1/0, where the numerals preceding and following the slash (/) denote the working conditions of the PIN diodes: “0” signifies the OFF state, and “1” indicates the ON state. Figure 3 illustrates the electric field distributions of the metasurface when the PIN diode switches states at 5 GHz, which further elucidates the intrinsic mechanism of polarization conversion of the metasurface. By switching the states of the PIN diode, the metasurface can convert the incident electromagnetic wave with *x*-polarization into the transmitted electromagnetic wave with *u*/*v*-polarization.

**Figure 4:**
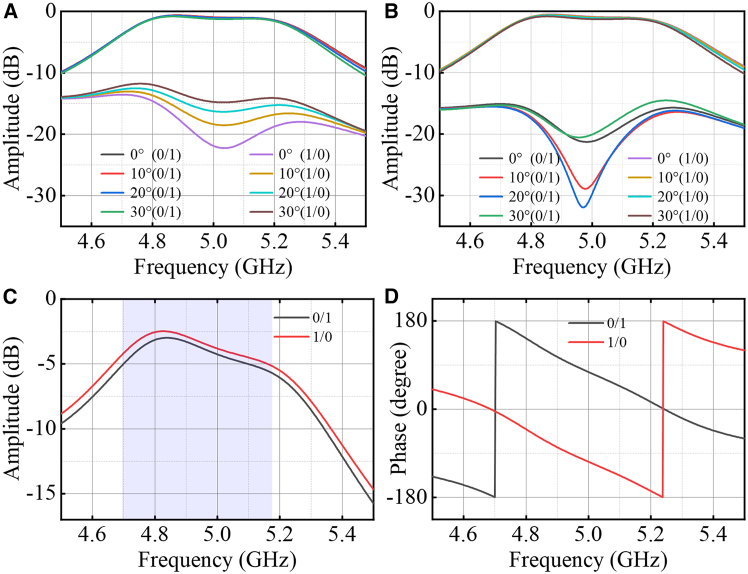
Simulation results (A) Amplitude of the received signal in u-polarization. (B) Amplitude of the received signal in v-polarization. (C) Amplitude of the received signal in y-polarization. (D) Phase of the received signal in y-polarization.

Due to the structural symmetry of the metasurface element, when receiving *y*-polarized signals, the amplitude of the received signal remains unchanged during PIN diode state switching, whereas a 180° phase difference is observed between the two states. Figures 4A and 4B illustrate the amplitude responses under *u*- and *v*-polarized reception conditions under oblique incidence. The simulation results demonstrate that the designed unit exhibits favorable angular stability, a high transmission coefficient, and a polarization isolation of 15 dB. Figures 4C and 4D present the amplitude and phase characteristics for *y*-polarized reception, respectively. Compared to the *u*-polarized amplitude, the *y*-polarized amplitude is reduced by 3 dB. When the PIN diode state is toggled at 5 GHz, the amplitude remains nearly constant, while a distinct 180° phase shift is achieved between the two states. Therefore, the proposed ±45° polarization conversion metasurface can independently control the amplitude and phase of the electromagnetic wave in both the *u*-polarized (or *v*-polarized) and *y*-polarized directions, which forms the basis for achieving BASK modulation and BPSK modulation with the metasurface.

### The concept of backscatter communication

**Figure 5:**
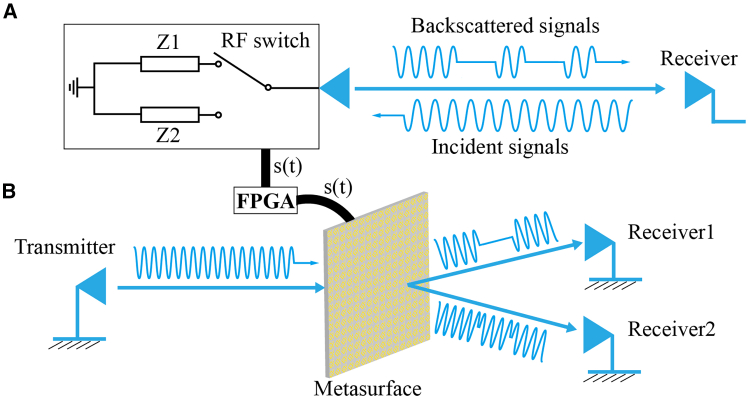
System architecture diagrams (A) Traditional backscatter communication architecture. (B) Backscatter communication architecture based on IMS.

The concept of backscatter communication based on a metasurface is rooted in the principles of traditional BC. As shown in Figure 5A, in conventional BC systems, information encoding of “0” and “1” is achieved through switching between the antenna’s reflective and non-reflective states. Specifically, this state regulation is realized by toggling the antenna’s load impedance via an RF switch integrated into the feed circuit. When the RF switch connects to different loads, the antenna’s reflection coefficient undergoes significant changes, thus modulating the incident wave. The RF switch is typically controlled by a low-power controller (e.g., FPGA or microcontroller), with the control signal s(t) generated according to sensor feedback data.

Figure 5B shows that our system can perform two types of modulation on the incident wave. Under these two modulation scenarios, the digital signal, denoted as y[n], which is obtained after the information received by the receiving antenna is converted by the ADC (Analog-to-Digital Converter), is expressed as follows:

(Equation 4) y[n]=x[n]+αB[n]x[n]+w[n]

(x[n]) is the digital sampling of the radio frequency carrier signal source, (w[n]) is the additive noise of the receiver, α represents the complex attenuation coefficient of the backscattered signal, and B[n] is a binary symbol sequence representing the transmitted data, which is used to carry the data information to be transmitted. Its value is determined by the modulation method. For amplitude modulation, BASK, B[n]∈{0,1}. When B[n] = 0, the polarization direction of the receiving antenna is orthogonal to that of the transmitted wave, and the amplitude of the received signal is low. When B[n] = 1, the polarization direction of the receiving antenna is the same as that of the transmitted wave, and the amplitude of the received signal is high. For phase modulation BPSK, B[n]∈{+1,−1}, which represents the phase shift: when B[n] = +1, the phase of the reflected signal is the same as that of the original signal; when B[n] = −1, the phase of the reflected signal differs from that of the original signal by π. By averaging the N digital samples of the receiver, the average power of the received signal can be obtained, as shown in later in discussion^8^:

(Equation 5) $$\frac{1}{N}\sum_{n=1}^{N}|y\left[n\right]{|}^{2}=\frac{1}{N}\sum_{n=1}^{N}|x\left[n\right]+\alpha B\left[n\right]x\left[n\right]+w\left[n\right]{|}^{2}$$

If the received noise is ignored, the above equation can be rewritten as:

(Equation 6) $$\frac{1}{N}\sum_{n=1}^{N}|y\left[n\right]{|}^{2}=\frac{1}{N}\sum_{n=1}^{N}|x\left[n\right]+\alpha B\left[n\right]x\left[n\right]{|}^{2}=\frac{1}{N}\sum_{n=1}^{N}|x\left[n\right]{|}^{2}\cdot {\left|1+\alpha B\left[n\right]\right|}^{2}$$

If $P=\frac{1}{N}\sum_{n=1}^{N}|x\left[n\right]{|}^{2}$ represents the average power directly from the carrier signal. For BASK modulation, when B[n] is equal to 0 or 1, the average carrier power at the receiver is *P* and |1+α|^2^*P*. The receiver must be able to distinguish between *P* and |1+α|^2^*P* in order to demodulate the information. For BPSK modulation, when B[n] is equal to 1 or -1, the average carrier power at the receiver is |1+α|^2^*P* and |1-α|^2^*P*. The receiving end can recover the data by comparing the differences in carrier power.

### Experimental verification

**Figure 6:**
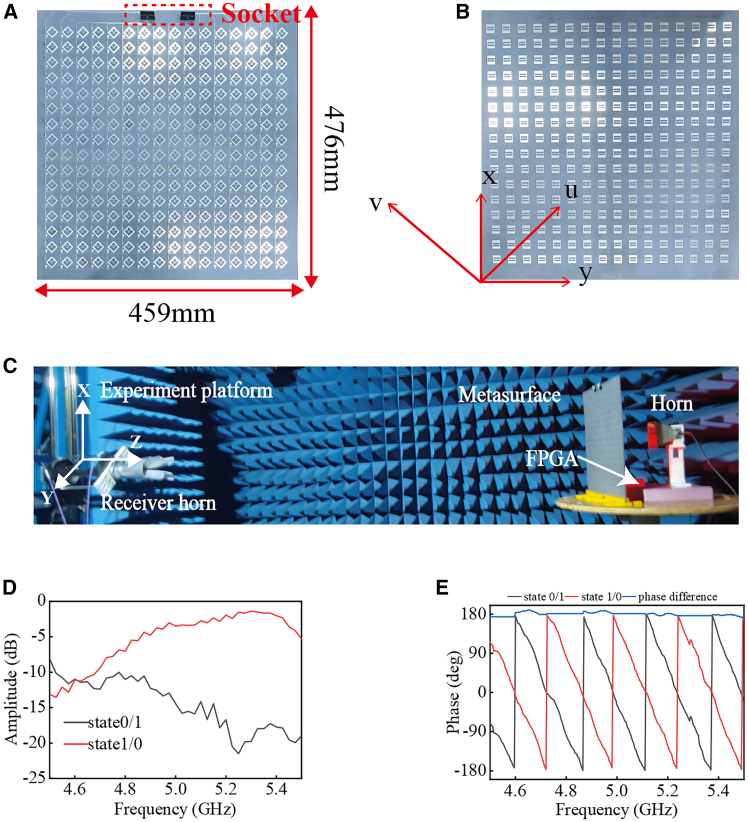
Prototype and measurement results of the IMS (A) Front-surface photograph of the IMS. (B) Back-surface photograph of the IMS. (C) Photograph of the anechoic chamber test setup. (D) Amplitude test results for the receiving antenna in v-polarization. (E) Phase test results for the receiving antenna in y-polarization.

As shown in Figure 6A and 6B, a 16 × 16 IMS array was fabricated. Each atom in the upper layer of the metasurface requires the soldering of two PIN diodes. The bias lines for each metasurface column are tied together to share a common bias voltage, connected to sockets, and then linked to the FPGA via a ribbon cable. To evaluate the performance of the metasurface, we conducted a transmission test in a microwave anechoic chamber. As shown in Figure 6C, the transmitting horn is in y-polarization, and the receiving horn is in u-polarization. The transmitting horn is 0.5 m away from the metasurface, and the receiving horn is 3 m away from the metasurface. Figure 6D shows that the trends of the test results are the same as those of the simulation results. However, due to the errors in the processing and the equivalent parameters of the PIN diodes, there is a slight shift in the central frequency. Figure 6E shows the phase curve when the PIN diodes are changed while the receiving horn is in *y* polarization. The simulation results are consistent with the test results, indicating that the metasurface can perform both amplitude modulation and phase modulation, forming the basis for subsequent information encoding. Before starting the information modulation, we tested the interference of the experimental environment on the communication. As shown in Figure S1A (supporting information), the transmitting antenna is connected to the signal source, and the receiving antenna is connected to the spectrum mode of the vector network analyzer. By switching the states of the PIN diodes and changing the distance of the receiver, we obtained the graph of the received power changing with the distance, as shown in Figure S1B (supporting information). As the distance increases and the transmitting power weakens, the polarization isolation will decrease, which will lead to a reduction in the signal-to-noise ratio and impair the distinction between the two information states 0 and 1.

**Figure 7:**
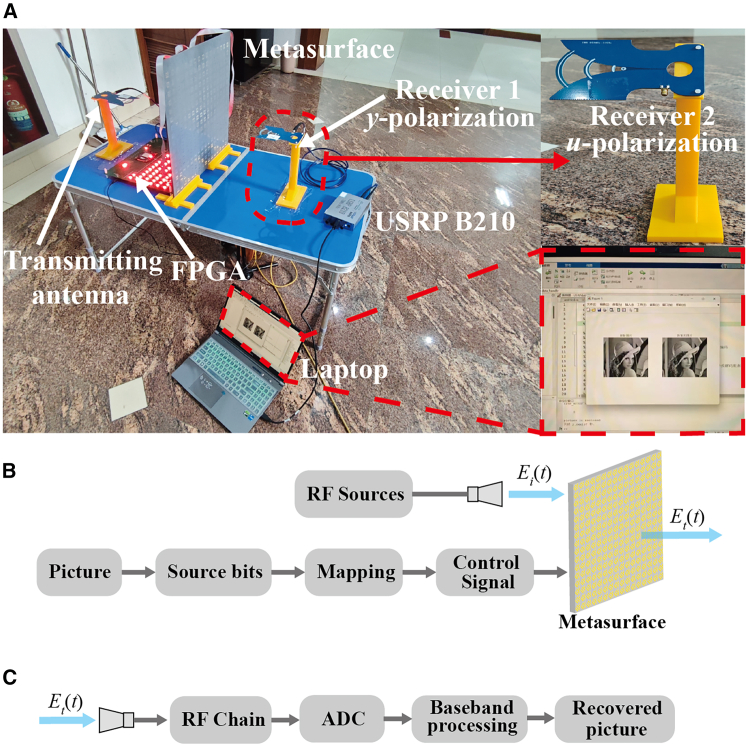
Experimental scenario and signal processing flow of polarization-diversity backscatter communication (A) Photograph of the IMS-based polarization-diversity backscatter communication test setup. (B) Block diagram of signal processing at the transmitter. (C) Block diagram of signal processing at the receiver.

**Figure 8:**
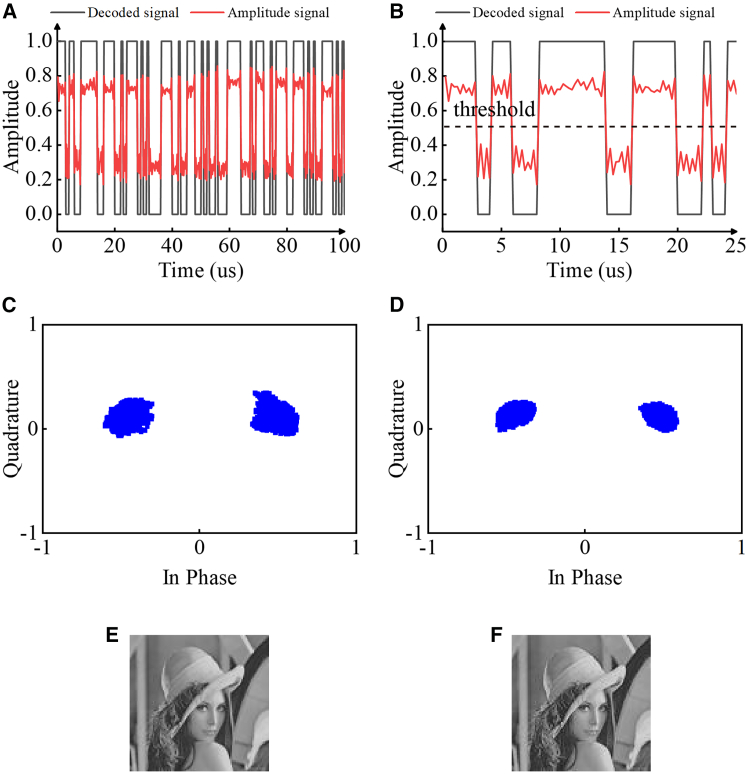
Experimental results of polarization-diversity backscatter communication (A) Received and decoded signals with BASK modulation. (B) Detailed diagram of the 0–25 μs segment in (A). (C) BPSK constellation diagram at 1 Mbps symbol rate. (D) BPSK constellation diagram at 2 Mbps symbol rate. (E) Original image. (F) Received image.

The test diagram of the communication system is presented in Figure 7A. The receiving antenna can be placed along the ±45° polarization direction (BASK modulation) or the *y* - polarization direction (BPSK modulation). In this test, we choose the *y* - polarization direction as an example. The bitstreams of the image information are stored in the FPGA. When the control pin of the FPGA is at +5 V, the working states of all PIN diodes on the metasurface are 1/0, and at this time, the transmitted wave is in 45° polarization. Conversely, when the voltage of the FPGA control pin is −5 V, the transmitted wave is in −45° polarization. The transmitting antenna and the receiving antenna are respectively connected to the transmitting (TX) port and the receiving (RX) port of the software-defined radio device USRP B210. It should be noted that the transmitting antenna emits an unmodulated carrier signal at 5 GHz, allowing it to be connected to an independent signal source. The detailed experiment is shown in Figure 1, Figure 2, Figure 3, Figure 4, Figure 5, Figure 6, Figure 7, Figure 8 (supporting information). During information transmission, the FPGA periodically switches states (receiver 1 in y-polarization and receiver 2 in u-polarization). According to the simulation results of the unit, the phase of receiver 1 will change periodically, and the amplitude of receiver 2 will also change periodically. After digital sampling and symbol discrimination inside the USRP, the binary symbols 0 and 1 are decoded, as shown in Figures 7B and 7C. To accurately and error-free recover the image information, a start frame needs to be added to the original image encoding. During the decoding process, the starting position of the image bitstreams is found based on the start frame. Subsequently, the complete image bitstreams are obtained according to the image pixel size, and then the binary bitstreams is restored to an image through MATLAB software.

The results of the communication experiment are shown in Figure 8, which presents the test results of BASK and BPSK. The maximum distortion-free communication rate of ASK can reach 1 Mbps, as depicted in Figures 8A and 8B. During the experiment, the amplitude threshold was set to 0.5, where amplitudes exceeding 0.5 are quantized to ‘1’ and those below 0.5 to ‘0’. The maximum distortion-free communication rate of BPSK can reach 2 Mbps. It is worth noting that the communication rate is determined by the FPGA operating frequency. As the FPGA frequency increases, severe waveform distortion occurs on the PIN diode, which significantly limits the communication rate of the backscatter communication system, as shown in Figure S3 (supporting information). This is attributed to the fact that BPSK possesses strong anti-noise capability. Since it conveys information by altering the phase, the phase variation is less sensitive to noise and performs favorably in environments with a low signal-to-noise ratio. In contrast, BASK has relatively poor anti-interference capability. As BASK relies on the variation of amplitude, and the amplitude is significantly affected by noise, the signal is prone to distortion, resulting in inferior performance under low signal-to-noise ratios. The transmitting antenna is positioned 0.5 m away from the metasurface, and the receiving antenna is also 0.5 m distant from the metasurface. The constellation diagram results of the BPSK tests at two different symbol rates are illustrated in Figures 8C and 8D). The constellation diagrams converge to two points, indicating a good signal-to-noise ratio. The images obtained upon decoding the two modulation schemes are nearly indistinguishable from the original ones, and the bit error rate (BER) is on the order of 1 × 10^−5^, thus achieving satisfactory communication performance, as shown in Figure 8F. In addition, we simultaneously receive information from two polarization channels within a ±30° range, and the experimental results show good performance, as shown in Figures S2B–S2E (supporting information).

It is worth noting that the carriers in both experiments are emitted by the signal source. Therefore, in theory, the carrier can also be the existing WIFI signal in the environment. By using the metasurface to perform secondary modulation on the WIFI signal to transmit information, compared with the transmitting end architecture of the traditional communication system, it avoids the use of power amplifiers, oscillators, and digital modulators, which greatly reduces the communication power and simplifies the architecture of the communication system. It has the advantages of energy conservation and simple architecture. In addition, backscatter communication is also a form of secure communication. Only the users on the receiving side of the metasurface are likely to receive the modulated carrier signal. And even if they receive the modulated carrier signal, they need to know the modulation method and the corresponding decoding strategy to recover the information. To illustrate the advantages of the proposed polarization diversity backscatter communication system based on information metasurfaces, we compare it with some other reported metasurfaces for wireless communication, and the specific results are shown in Table 1. With its polarization diversity design, the system supports two information modulation schemes (ASK and BPSK) and is compatible with different polarization directions. Meanwhile, compared with the reported schemes in Table 1, it features lower hardware complexity and higher communication rate, providing a highly promising platform for the application of metasurfaces in the field of wireless communication.

**Table 1:** Comparison of the proposed polarization diversity BC system based on IMS with other reported counterparts

Reference	Maximum Communication Rate	Modulation Scheme	Design Complexity	Number of Channels
43	500Kbps	ASK、BPSK、QPSK	Pin Diode	3
44	–	ASK	Pin Diode	1
45	1Mbps	QPSK	Varactor Diode	1
46	–	polarization modulation (PoM)	Varactor Diode, Polarization Discrimination Antenna	1
47	234.375Kbps	BASK、BPSK、QPSK、8PSK、8QAM、16QAM	Varactor Diode, Amplifier, Filter, and Energy Management Circuit	1
This work	2Mbps	ASK、BPSK	Pin Diode	2

## Discussion

We propose a polarization-diversity backscatter communication system based on an intelligent metasurface (IMS) operating at 5 GHz, which supports backscatter communication with two modulation schemes (BASK and BPSK) in two polarization directions. The polarization conversion mechanism can reduce interference from the direct link between the transmitter and receiver and improve the signal-to-noise ratio (SNR). Furthermore, compared with the traditional cross-polarization method, the ±45° polarization scheme exhibits enhanced resistance to interference from the multipath effect. Numerical and experimental results demonstrate that the system can transmit a 98 × 102-pixel image without distortion under both BASK and BPSK modulation. In addition, the scenario where the receiver is within a ±30° range was also investigated. Experimental results show that the receiver can reliably receive information over this angular range. Our polarization-diversity backscatter communication system enhances the reliability and stability of backscatter communication, thus holding significant potential for future IoT applications.

### Limitations of the study

Although this study represents an advancement in metasurface-based backscatter communication, several limitations should be noted. First, the maximum communication rate of 2 Mbps is limited by the FPGA operating frequency and the switching speed of the PIN diodes, which constrains its applicability in high-speed scenarios. Second, the system was primarily evaluated over short distances (with the transmitter placed 0.5 m away and the receiver 3 m from the metasurface). In long-range deployments, polarization isolation and signal-to-noise ratio tend to deteriorate, while the 16 × 16 metasurface array also restricts signal propagation and coverage. Furthermore, the system remains stable only within a ±30° incidence angle range, indicating limited robustness against large-angle incidence and complex multi-directional interference in practical environments.

## Resource availability

### Lead contact

Further information and requests for resources and reagents should be directed to and will be fulfilled by the lead contact, Long Li (lilong@mail.xidian.edu.cn).

### Materials availability

This study did not generate new reagents.

### Data and code availability

• Data reported in this article will be shared by the lead contact upon request.
• This article does not report original code.
• Any additional information required to reanalyze the data reported in this article is available from the lead contact upon request.

## Acknowledgments

This work was supported by 10.13039/501100001809the National Natural Science Foundation of China (No. 62288101), 10.13039/501100012166the National Key Research and Development Program of China under Grant 2023YFB3811503, and the Fundamental Research Funds for the Central Universities and the Innovation Fund of 10.13039/501100005320Xidian University (No. 20103224952 and No. YJSJ23016).

## Author contributions

Conceptualization, G.L., X.M., and J.H.; methodology, G.L. and X.M.; software, G. L. and X.M.; validation, L.Z. and R.L.; writing – original draft preparation, G.L.; writing – review and editing, X.M. and J.H.; supervision, X.M., J.H., and L. Z.; project administration, H.L. and L.L.; funding acquisition, H.L. and L.L. All authors have read and agreed to the published version of the manuscript.

## Declaration of interests

The authors declare no competing interests.

## STAR★Methods

### Key resources table

**Table:** 

REAGENT or RESOURCE	SOURCE	IDENTIFIER
**Software and algorithms**		

Ansys Electronics 2022	Ansys	https://www.ansys.com/zh-cn/products/electronics
MATLAB R2023a	MathWorks	https://www.mathworks.com/products/new_products/release2023a.html
GNU Radio	GNU	https://www.gnuradio.org/

### Experimental model and study participant details

This study employs Ansys Electronics Desktop software to design and simulate the metasurface, and the backscatter communication model is implemented on the GNU Radio platform.

### Method details

#### Binary conversion of image data

The pixel grayscale values are converted into 8-bit binary sequences using MATLAB and exported into a binary text file, which encapsulates the complete image information. This data is then bit-mapped to FPGA control signals and stored in the FPGA’s memory units (e.g., ROM/RAM).

#### Recovery of experimental image data

The image data recovery process comprises two primary stages: first, the demodulated and quantized binary data is acquired at the receiver using GNU Radio; then, in MATLAB, the positioning guide codes are removed, the complete binary dataset is extracted based on the image pixel dimensions, and it is finally reorganized into a matrix matching the image pixels to reconstruct the original image.

### Quantification and statistical analysis

The simulation data is produced by Ansys Electronics software. Figures shown in the main text were produced by ORIGIN and Microsoft Visio from the raw data.

### Additional resources

Any additional information about the simulation and data reported in this paper is available from the lead contact on request.

## Supplemental information

Document S1. Figures S1–S4 and Notes S1–S3
