# Supplementary material for: Polarization-diversity backscatter communication based on programmable information metasurface
Source: iScience. 2025 Dec 30;29(2):114577. doi: 10.1016/j.isci.2025.114577 (PMC12829126; doi:10.1016/j.isci.2025.114577)
Supplement: Document S1. Figures S1–S4 and Notes S1–S3 [file mmc1.pdf]

iScience, Volume *29*

Supplemental information

Polarization-diversity backscatter communication based on programmable information metasurface

Guoliang Luo, Xiangjin Ma, Jiaqi Han, Lihao Zhu, Rui Li, Haixia Liu, and Long Li

Supporting Information

Polarization-Diversity Backscatter Communication Based on Programmable Information Metasurface

Guoliang Luo, Xiangjin Ma, Jiaqi Han, Lihao Zhu, Rui Li, Haixia Liu, Long Li**

Key Laboratory of High-Speed Circuit Design and EMC of Ministry of Education, School of Electronic Engineering, Xidian University, Xi'an 710071, China

[*] Corresponding authors. E-mail: hxliu@xidian.edu.cn and lilong@mail.xidian.edu.cn

Note 1 Influence of distance on metasurface performance under complex environments

To investigate the communication distance of the backscatter communication system, a test environment was set up as shown in Figure S1a. The transmitting horn, connected to an RF signal source, was positioned 50 cm away from the metasurface with horizontally polarized incident waves. The receiving horn, linked to a vector network analyzer configured in spectrum analyzer mode, had its polarization oriented at 45° relative to the horizontal polarization and was placed at a distance d from the metasurface. The FPGA controlled all PIN diodes on the metasurface to operate in either the 0/1 state (ON) or 1/0 state (OFF). Experiments were conducted with the signal source output power set to two different values: 0 dBm and 10 dBm. The relationship between the power received by the receiving horn and the receiver distance d is shown in Figure S1b. As shown in the figure, as the distance d increases, the difference in received power between the ON and OFF states decreases significantly, converging towards the noise floor. This reduction in signal separation leads to ambiguity in distinguishing between the information symbols ‘0’ (corresponding to OFF state) and ‘1’ (corresponding to ON state). Increasing the input power P_{in} can alleviate this degradation by improving the signal-to-noise ratio (SNR) and thus enhancing the distinguishability of the symbols.

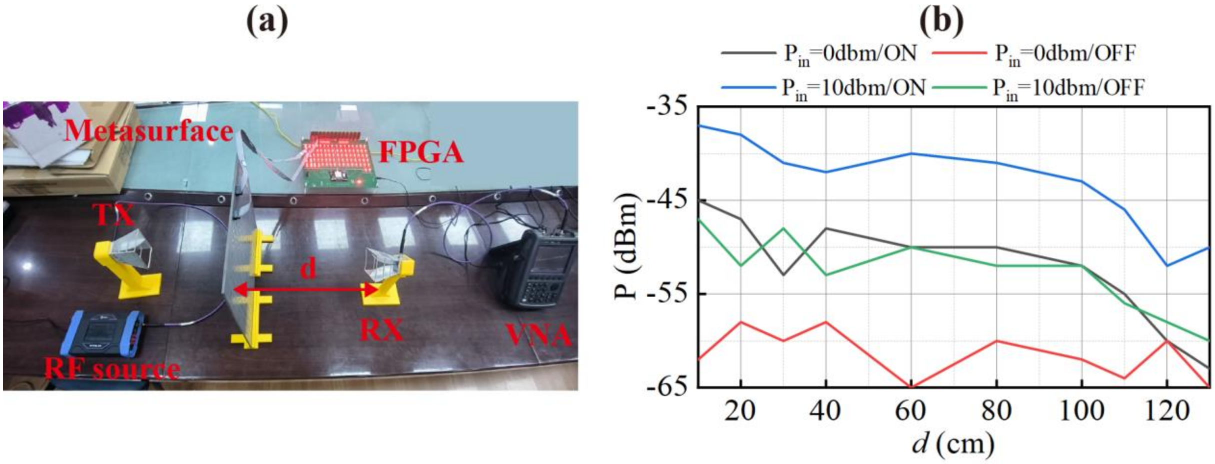

Figure S1. **a** Test Scenario Diagram. **b** Relationship between the received power and the distance from the receiver when the PIN diodes switch between two different states under different incident powers.

Note 2 Wide-angle polarization-diversity communication experiment

To demonstrate that our backscatter communication system can utilize existing electromagnetic waves in the environment for secondary modulation to transmit information, we connected the transmitting antenna to an RF signal source instead of the TX/RX ports of the USRP B210 (although the signal transmitted by the USRP B210 in the experiment was unmodulated), as shown in Figure S2a. During the unit simulation, it was verified that the designed unit exhibits angular stability within a ±30° range. The two receiving antennas are polarized differently, with receiving antenna 1 being horizontally polarized and receiving antenna 2 being polarized at a 45° angle. The transmitting and receiving ends are located on both sides of the metasurface, with minimal coupling interference between them, allowing the two polarization channels to simultaneously receive image information modulated by the metasurface. In the experiment, the transmitting antenna was positioned 45 cm from the metasurface, the receiving antennas were 40 cm from the metasurface, and the signal source output power was 0 dBm. The USRP B210 served as the receiver to capture information from the two polarization channels for processing. The demodulation method for receiver 1 was BPSK, and that for receiver 2 was BASK, with their receive gains set to 58 dB and 43 dB, respectively, in GNU Radio. We tested four relative positional states of the two receivers within the ±30° range, as shown in Figures S2 (b)–(e). When θ_1 increased, the two symbol point clusters in the constellation diagram moved closer to each other, but no divergence or overlap of the constellation diagram occurred within the ±30° range. This indicates that our polarization-diversity backscatter communication system possesses excellent angular stability.

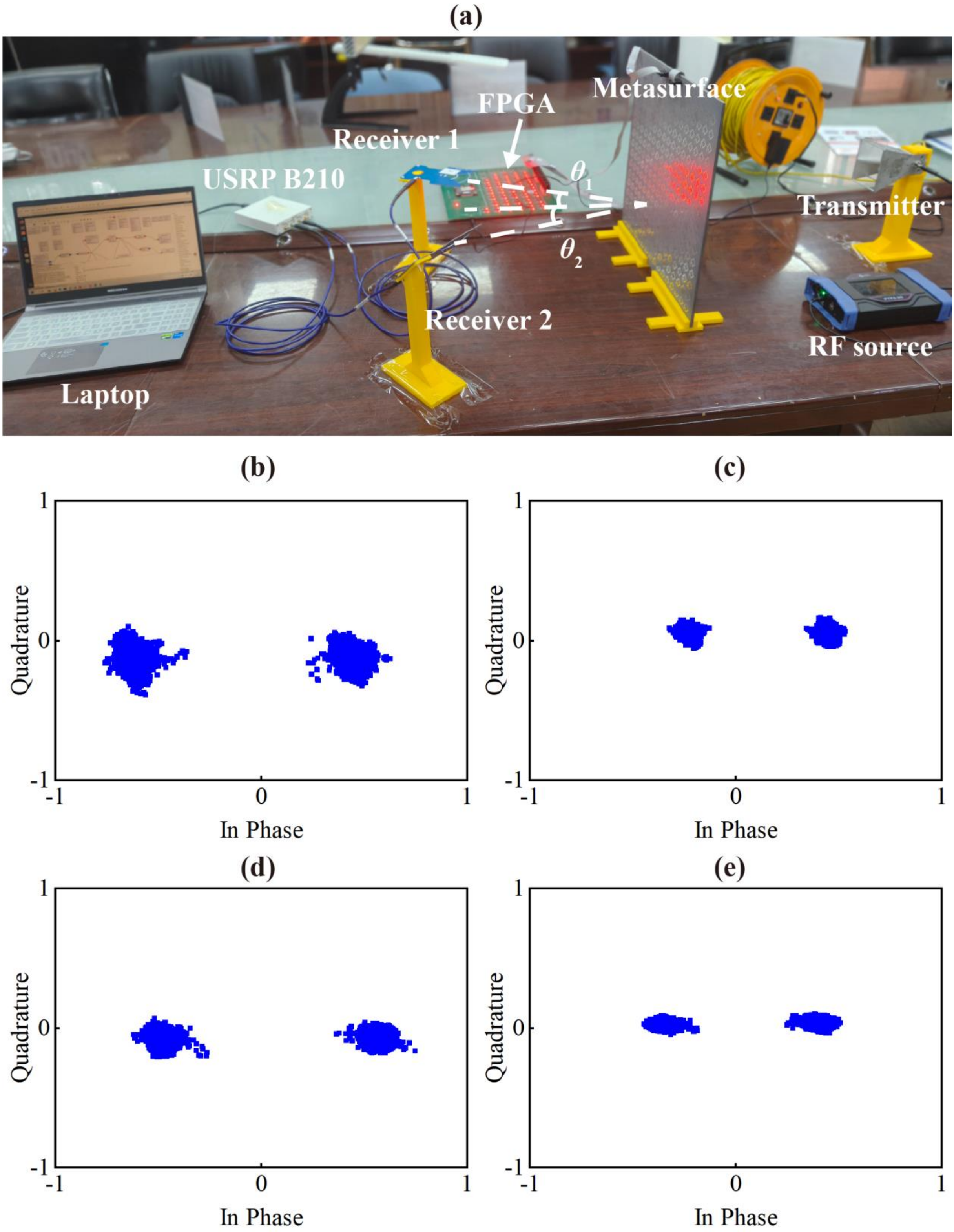

Figure S2. Scene diagram and test results of the wide-angle polarization-diversity communication experiment. a Experimental scene diagram. b Constellation diagram when $\theta_1=0°$, $\theta_2=15°$. c Constellation diagram when $\theta_1=30°$, $\theta_2=30°$. d Constellation diagram when $\theta_1=0°$, $\theta_2=0°$. e Constellation diagram when $\theta_1=15°$, $\theta_2=30°$.

Note 3. Influencing factors of communication rate

In this backscatter communication experiment, the maximum achievable communication rate of the system was limited to 2Mbps, primarily due to the signal switching rate. Figure S3 illustrates the block diagram of the circuit for generating ±5V signals. The DG333A single-pole double-throw analog switch chip, a key component in this circuit, has a maximum switching frequency of 5.7MHz. This chip is responsible for converting the 0/3.3V signals output by the FPGA into ±5V signals. During the experiment, a significant change in circuit performance was observed when 16 PIN diodes were connected in parallel at the output of each DG333A channel. Due to the cumulative capacitance effect of the PIN diodes, the square wave signal with a frequency of 5MHz applied to the PIN diodes suffered severe distortion. However, when the frequency of the square wave signal was reduced below 2MHz, the waveform on the PIN diodes remained normal, which is consistent with the results shown in Figure S4.

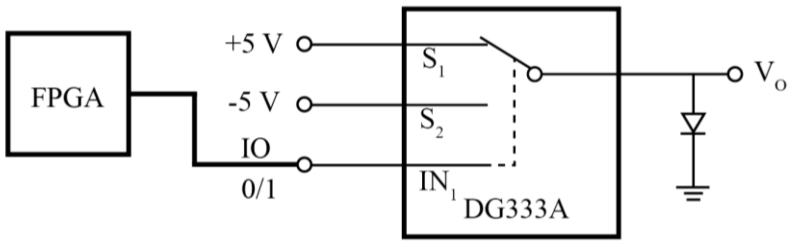

Figure S3. Block diagram of the ±5V generation circuit

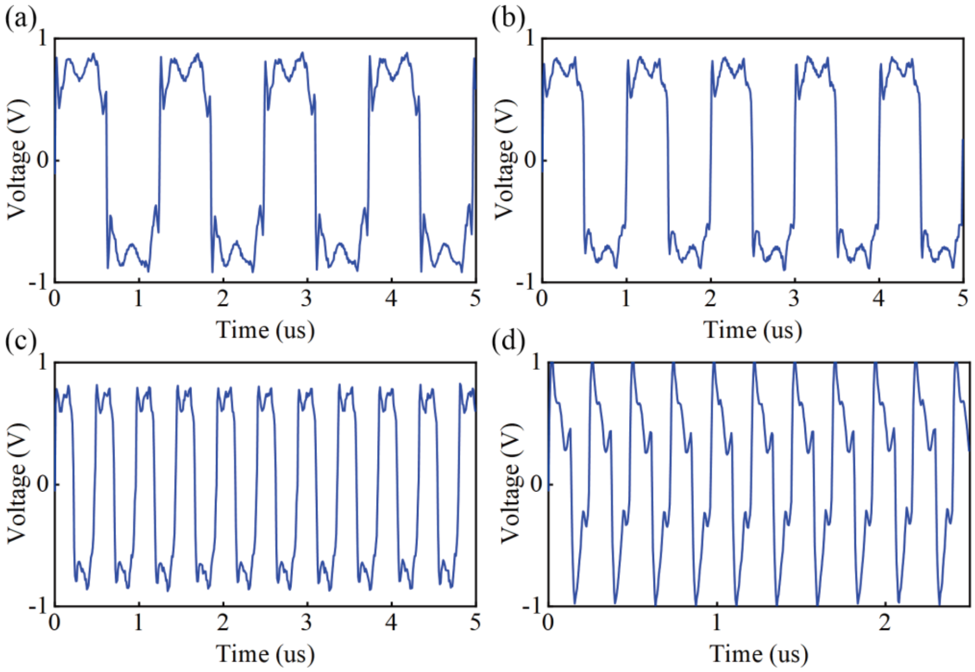

Figure S4. The measured switching speed of the metasurface sample. a-c The metasurfaces work normally when driven by square waves at 800 kHz, 1 MHz, and 2 MHz, respectively. **d** The effect deteriorates and waveform distortion occurs when the frequency is 5 MHz
